# The Role of Nitric Oxide in HSV-1 Infection: The Use of an Inducible Nitric Synthase Inhibitor Aminoguanidine to Treat Neuroinflammation

**DOI:** 10.3390/microorganisms13102222

**Published:** 2025-09-23

**Authors:** Magdalena Patrycy, Martyna Janicka, Agnieszka Kauc, Aleksandra Osińska, Małgorzata Antos-Bielska, Klaudia Bylińska, Oliwia Obuch-Woszczatyńska, Paweł Szymański, Marcin Chodkowski, Małgorzata Krzyżowska

**Affiliations:** 1 Division of Medical and Environmental Microbiology, Military Institute of Hygiene and Epidemiology, 01-163 Warsaw, Poland; magdalena.patrycy@wihe.pl (M.P.); martyna.janicka@wihe.pl (M.J.); agnieszka.kauc@wihe.pl (A.K.); aleksandra.osinska@wihe.pl (A.O.); malgorzata.bielska@wihe.pl (M.A.-B.); marcin.chodkowski@wihe.pl (M.C.); 2 Division of Microbiology, Department of Preclinical Sciences, Institute of Veterinary Medicine, Warsaw University of Life Sciences, 02-786 Warsaw, Poland; 3 Laboratory of Parasitology, Military Institute of Hygiene and Epidemiology, 01-163 Warsaw, Poland; klaudia.bylinska@wihe.pl (K.B.); oliwia.obuch@wihe.pl (O.O.-W.); 4 Division of Pharmacology and Toxicology, Department of Preclinical Sciences, Institute of Veterinary Medicine, Warsaw University of Life Sciences, 02-786 Warsaw, Poland; 5 Department of Pharmaceutical Chemistry, Drug Analysis and Radiopharmacy, Medical University of Lodz, 90-151 Lodz, Poland; pawel.szymanski@wihe.pl; 6 Department of Radiobiology and Radiation Protection, Military Institute of Hygiene and Epidemiology, 01-163 Warsaw, Poland; 7 Department of Pharmaceutical Chemistry and Biomaterials, Faculty of Pharmacy, Warsaw Medical University, 02-097 Warsaw, Poland

**Keywords:** nitric oxide, HSV-1, aminoguanidine, neuroinflammation, beta amyloid

## Abstract

Herpes simplex virus-1 (HSV-1) is a neurotropic virus that can infect the brain, and an uncontrolled infection can lead to severe encephalitis. NO can exert both antiviral as well as cytotoxic effects in the central nervous system (CNS) depending on its concentration and site of infection. In this study, we report that treatment of an intranasal murine HSV-1 infection with aminoguanidine (AMG) decreases both neuroinflammation and neurodegeneration markers, but its positive effect depends on the time of treatment. Specifically, early treatment with AMG impaired the activation of microglia/monocytes, leading to decreased virus-specific antiviral response and higher viral titers in the brain. However, AMG treatment during the peak of brain infection significantly improved antiviral response, reduced inflammation and improved general clinical score. We also found that treatment with AMG decreased beta amyloid levels during both primary and latent infections and protected from the accumulation of phosphorylated Tau protein during early infection. Our findings position inducible nitric oxide synthetase (iNOS) as a potential therapeutic target for mitigating virus-induced neuroinflammation and neurodegeneration.

## 1. Introduction

Nitric oxide (NO) is a pivotal signaling molecule involved in a wide range of cellular and physiological processes, including vasodilation, neurotransmission and inflammatory response [1,2,3,4]. It is generated in various cell types through the conversion of L-arginine to L-citrulline by three distinct isoforms of NO synthase (NOS). Neuronal NOS-I (nNOS) and endothelial NOS-III (eNOS) are constitutively expressed and produce NO only for short periods of time (seconds to minutes), while NOS-II is an inducible form (iNOS) that produces 10–100 times more NO for hours to days following infection and inflammation [1,2,3,4]. iNOS expression is localized in several tissues and cells, including hepatocytes, lung epithelial cells, macrophages, neutrophils, monocytes and endothelial cells [1,2,3,4].

The antiviral activity of NO has been contributed to its activity as a free radical: its reaction with superoxide O2^•−^, one of several reactive oxygen species (ROS), yields peroxynitrite (ONOO•/HOONO), a short-lived radical that induces oxidation, nitration and S-nitrosylation of proteins [5,6,7]. As a direct antiviral effect, S-nitrosylation targets thiol moieties of cysteine-containing viral enzymes such as proteases, ribonucleotide reductases and reverse transcriptases, as well as nucleocapsid proteins [6,7,8]. It is believed that NO production may provide a first line of innate immune defense against viruses causing respiratory infections (human rhinovirus (HRV), influenza A virus (IAV) and severe acute respiratory syndrome coronavirus (SARS-CoV-2)) [8,9,10,11,12]. However, for non-respiratory infections, excessive and chronic production of NO may contribute to the pathogenesis of viral infection [13,14]. Herpes simplex virus type 1 (HSV-1) is a human pathogen with a high prevalence (64% of the global population). Most people become HSV-1 infected early in life through orolabial mucosa, and the outcome of infection with HSV-1 can be asymptomatic, mild or life threatening [15]. People infected with HSV can expect to have several (typically four or five) outbreaks (symptomatic recurrences) within a year.

Herpes simplex encephalitis (HSE), which predominantly affects children and the elderly, is one of the most common forms of viral encephalitis and has remarkably poor outcomes despite the availability of good antiviral therapy. More than 70% of patients without treatment die, and most patients subsequently suffer from epilepsy, mental retardation and chronic neuronal deficits; only 2–5% of patients recover completely [15,16]. Many studies have strongly supported the concept that repeated reactivations of HSV-1 may lead to persistent neuroinflammation, the formation of amyloid plaques and neurofibrillary tangles and, eventually, Alzheimer’s disease (AD) [17,18,19,20,21,22]. Therefore, there is a constant need to search for substances that can help to decrease excessive inflammation during HSE, without disturbing the development of a specific anti-HSV-1 immune response.

Mechanisms by which HSV-1 may cause neurodegeneration include the induction of Aβ accumulation [17,18], tau phosphorylation [19], inflammation [20], DNA damage, neuronal cell death [21], microglial overactivation and impaired Aβ clearance [21,22]. It has been also suggested that Aβ aggregation may act as an innate immune response to microbial infections, acting as a microbial peptide, though its exact mechanism of action remains unknown [22].

In response to HSV-1 infection, both resident and non-resident immune cells of the brain (microglia, macrophages and neutrophils) increase the production of ROS and NO, and the production of both contributes to the control of viral spread [15,23,24,25,26]. Several authors have demonstrated that the inhibition of iNOS activity early during HSV infection with aminoguanidine sulphate (AMG) leads to increased intranasal HSV-1 [27,28] and intravaginal herpes simplex virus type 2 (HSV-2) [29] infection and inflammation. AMG is also a potent antioxidant compound and free radical scavenger able to cross the blood–brain barrier (BBB) [30]. Therefore, its ability to cross the BBB can be used as an anti-inflammatory agent.

We have previously demonstrated the existence of a link between the dose of NO and type of glial cell and its influence on HSV-1 replication and inflammatory reaction in vitro [31]. Furthermore, we also observed co-localization of NO production with the accumulation of β-amyloid peptide in HSV-1-infected neurons in vitro. Generally, an NO donor increased the accumulation of β-amyloid in uninfected primary neuronal cultures, while the NO inhibitor (AMG) decreased its accumulation in HSV-1-infected neuronal cultures [31].

Considering the dual role of NO in viral infections and the role of excessive neuroinflammation in HSE and further sequelae, the aim of this work was to determine (a) the influence of iNOS inhibition by AMG and (b) its timing on the antiviral immune response and neuroinflammation in the murine model of HSV-1-induced encephalitis. Additionally, we assessed the possible influence of AMG on neuroinflammation-related neurodegeneration during early and latent infection.

## 2. Materials and Methods

### 2.1. Virus and Cells

HSV-1 (strain ID 2762) isolated from a patient with herpesvirus encephalitis (kindly provided by Professor Thomas Bergström, Department of Virology, University of Gothenburg) was grown and titrated in Vero cells (ATCC^®^ CCL-81, ATCC, Washington, DC, USA) and kept at −80 °C until use. The virus was diluted in minimum essential medium (MEM) (Thermo Fisher Scientific, Waltham, MA, USA), kept on ice and administered to mice within one hour. Vero cells, C8-D1A astrocytes (ATCC^®^ CRL-2541, ATCC) and RAW-Dual™ cells (InvivoGen, Toulouse, France) were maintained in Dulbecco’s modified Eagle’s medium with GlutaMAX (DMEM) supplemented with 10% fetal bovine serum (FBS), 100 units/mL penicillin and 100 µg/mL streptomycin (Thermo Fisher Scientific). Every second split, Zeocin^®^ (200 μg/mL) (InvivoGen) was added to RAW-Dual™ cells to maintain the secreted embryonic alkaline phosphatase (SEAP) activity (NF-κB activation) and Lucia luciferase (activation of interferon regulatory factors, IRFs). The supernatants were used to detect SEAP activity with QUANTI-Blue™ Solution and Lucia luciferase with QUANTI-Luc™ 4 Lucia/Gaussia detection reagent (InvivoGen).

### 2.2. Mice and Infection

Male and female (50:50) 6- to 8-week-old C57BL/6 mice were purchased from the Centre of Experimental Medicine in Medical Academy of Białystok (Białystok, Poland). Size of an animal group was calculated on the basis of an alpha parameter < 0.05 and the power of the statistical tests used (0.8). Before the experiment, mice were randomly divided into groups of mixed sexes. All treatment groups were blinded. For intranasal infection with HSV-1, mice were anesthetized with isoflurane (Baxter, Tramco, Wolskie, Poland), and a total dose of 1 × 10^6^ PFU of HSV-1 in 10 μL was given intranasally. Aminoguanidine hemisulfate salt (Sigma; St. Louis, MO, USA) was dissolved in NaCl and administered at a dosage of 400 mg/kg daily of an intraperitoneal treatment. Animals were monitored each day for the signs of illness, as described before [28]. On days 3, 7 and 60 post-infection (p.i.), mice were sacrificed by cervical dislocation, and tissues were collected for further tests. Brains intended for cryosections underwent transcardial perfusion with cold PBS under anesthesia with ketamine hydrochloride and xylazine (Vetoquinol, Gorzow, Poland).

### 2.3. Flow Cytometry Analysis

Single-cell suspensions were prepared from the brains and trigeminal ganglia of HSV-1-infected and uninfected control mice, as described previously [32,33]. Cell suspensions were pre-treated with the Fc receptor-blocking rat anti-CD16/32 antibody (2.4G2) (BD Biosciences, Franklin Lakes, NJ, USA) according to the manufacturer’s protocol. The detailed list of antibodies used to detect T cells, NK cells, microglia, monocytes and dendritic cells is given in Supplementary Table S1. HSV-1-specific T cells were detected using the SSIEFARL-PE tetramer (Creative Biolabs, Shirley, NY, USA). Stained cells were acquired using CytoFLEX LX (Beckman Coulter, Warsaw, Poland) and analyzed using FlowJo software V10 (Tree Star, Ashland, OR, USA). The compensation matrix was calculated using antibody-capturing beads (VersaComp, Beckman Coulter, Warsaw, Poland) stained with single antibody conjugates; the details for staining are given in Supplementary Materials.

### 2.4. Quantitative PCR

Total DNA and RNA from the brains and trigeminal ganglia were isolated using the RNA/DNA Extracol kit (Eurx, Gdansk, Poland), according to the manufacturer’s instructions. Titration of HSV-1 was performed by qPCR with primers and probes for the viral envelope glycoprotein (gB), as described previously [32,33], in the QuantStudio™ 5 Real-Time PCR System (Thermo Fisher Scientific) with GoTaq^®^ Probe qPCR Master Mix (Promega, Madison, WI, USA). Data are expressed as the HSV-1 copy number per ng of the total DNA in the tissue. The expression of lytic/latency-related genes, namely immediate early genes ICP0 and ICP27, a leaky-late gene (gB) and latency-associated transcript (LAT) was measured as described by Menendez et al. [34]. HSV-1 LAT and lytic genes were normalized to the mean threshold cycle (CT) of the β-actin housekeeping gene.

Quantitative PCR of cytokines and chemokines was performed using GoTaq^®^ Probe qPCR Master Mix (Promega, Madison, WI, USA) and TaqMan^®^ probes (Thermo Fisher Scientific) for the detection of IL-1β (Mm00434228_m1), IFN-γ (Mm01168134_m1), IFN-α4 (Mm00833969_s1), TNF-α (Mm00443258_m1), CXCL9 (Mm00434946_m1), CXCL10 (Mm00445235_m1), IL-10 (Mm01288386_m1), IL-6 (Mm00446190_m1) and GADPH (Mm99999915_g1), according to the manufacturer’s instructions in the QuantStudio™ 5 Real-Time PCR System (Thermo Fisher Scientific). Results were analyzed with the 2^−ΔΔCt^ method (2^−ΔΔCt^).

### 2.5. Confocal Microscopy

After overnight fixation in 4% paraformaldehyde/PBS, brains were saturated with 30%/PBS sucrose, frozen in liquid nitrogen and cut into 10 μm cryostat sections. Sections were stained overnight at 4 °C with the following primary antibodies diluted in working solution (2% bovine serum albumin (BSA) and 0.1% saponin in PBS): rabbit polyclonal anti-HSV-1/2 (Dako, Agilent, Santa Clara, CA, USA), rat monoclonal anti-CD11b-APC (clone M1/70, BD Biosciences), polyclonal goat anti-IBA1 (Thermo Fisher Scientific), anti-GFAP-Alexa Fluor^®^647 (clone 2A5, Abcam, Symbios, Gdansk, Poland) and anti-iNOS-PE (CXNFT, eBioscience, San Diego, CA, USA). For the detection of primary antibodies, Alexa Fluor^®^ 555 or 647 anti-goat polyclonal antibodies (Thermo Fisher Scientific) were used. Slides were mounted in ProLong Gold Antifade Reagent (Thermo Fisher Scientific) with 4′,6-diamidino-2-phenylindole (DAPI). Images were acquired using the Leica SP8 resonant scanning confocal system (Leica Microsystem, Wetzlar, Germany).

### 2.6. Primary Cultures

Mixed glial cultures were obtained as described by Krzyzowska et al. [32]. In short, the whole brains of neonatal C57BL/6 mice were digested with 0.25% trypsin/DMEM (Thermo Fisher Scientific) for 10 min. After enzymatic digestion, pieces were washed and resuspended in Dulbecco’s modified Eagle’s/F12 medium with GlutaMAX (DMEM/F12) supplemented with 10% FBS, 100 units/mL penicillin and 100 µg/mL streptomycin (Thermo Fisher Scientific). After 48 h, 5 ng/mL of murine recombinant granulocyte and macrophage colony-stimulating factor (GM-CSF) (Thermo Fisher Scientific) was added to medium. After reaching confluence (2–3 weeks), mixed glial cultures were used for further experiments.

### 2.7. ELISA

Brain homogenates were obtained by extraction with cold 5 M guanidine-HCl/50 mM Tris(hydroxymethyl)aminomethane (Tris) and diluted with 1× protease inhibitor cocktail (Pefabloc, Sigma-Aldrich, Poznan, Poland). Next, the homogenates were subjected to β-amyloid 1–42 and Tau (Phospho) [pS199] quantification using Mouse Amyloid Beta 42 (Ms Abeta 42) (Thermo Fisher Scientific) and Mouse Tau (Phospho) [pS199] (Thermo Fisher Scientific) ELISA tests according to the manufacturer’s protocol.

### 2.8. Statistics

For statistical analysis, GraphPad Prism version 7 (GraphPad software) was used. The data were subjected to the Shapiro–Wilk test for normality and Levene’s test for equality of variances. For data with non-Gaussian distributions, the nonparametric Wilcoxon test for dependent samples and Kruskal–Wallis test with post hoc multiple comparisons for comparison of all pairs were applied. The results are reported as the mean ± standard error of the mean (SEM) unless indicated otherwise. Here, *p* < 0.05 was considered statistically significant.

## 3. Results

### 3.1. Expression of iNOS Accompanies Neuroinflammation

**Figure 1:**
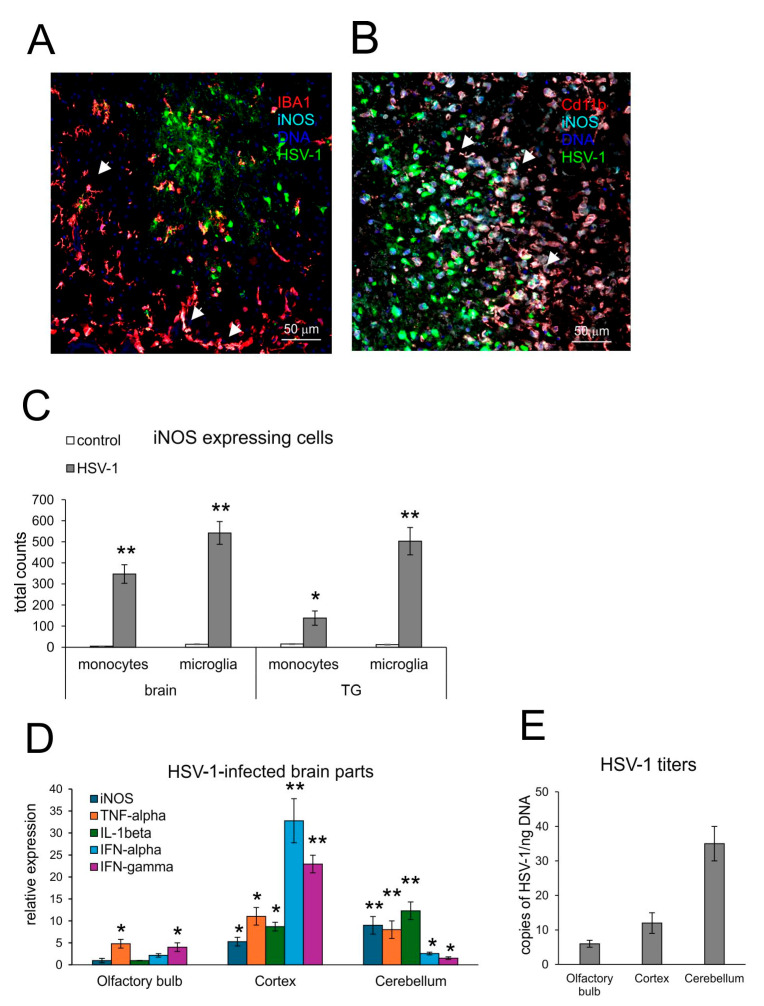
Expression of iNOS accompanies neuroinflammation in HSV-1-infected C57BL/6 mice at 7 d p.i. (**A**,**B**) Co-immunofluorescent staining for HSV-1 antigens (green), CD11b+ monocytes (red) or IBA-1+ microglia (red) and iNOS (turquoise) in the brain at 7 days post-infection (p.i.). Magnification × 200. White arrows indicate double-positive cells for lineage marker (IBA-1 or CD11b) and iNOS+. (**C**) Total counts of iNOS+ monocytes and microglia in brain and TGs at 7 d p.i. (**D**) Inducible NO synthetase (iNOS), antiviral interferons (IFN-α,γ) and inflammatory cytokines (TNF-α and IL-1β) in different brain parts (olfactory bulb, cortex and cerebellum) were analyzed by qPCR. (**E**) HSV-1 gB titers (copies/ng DNA) measured in the brain parts at 7 d p.i. by qPCR. C57BL6 mice were infected intranasally with HSV-1, and at day 7, brains and trigeminal ganglia (TGs) were collected for immunohistochemistry, flow cytometry analysis of the immune cells and isolation of DNA and RNA. Results are expressed as the mean ± SEM for N = 7. * represents significant differences with *p* ≤ 0.05, and ** means *p* ≤ 0.01, in comparison to the control (uninfected tissues).

Activated resident microglia and infiltrating monocytes have been shown to be the main contributors of brain inflammation in HSV-1 infection [23,24]. The excessive inflammatory response of microglia and monocytes is the main source of brain destruction in viral encephalitis [25]. However, early activation of microglia is also necessary to induce the innate response to HSV-1 in a paracrine manner by astrocytes [23,24]. Here, we analyzed the brain tissue and trigeminal ganglia (TGs) by confocal microscopy (Figure 1A) and flow cytometry (Figure 1B) for correlation between HSV-1 infection and iNOS expression. Confocal analysis of iNOS-positive cells showed the presence of iNOS+ microglia (Figure 1A) and monocytes (Figure 1B) surrounding the HSV-1 infectious foci in the brain, confirming the presence of NO-producing cells in HSV-1-induced neuroinflammation. Furthermore, iNOS-positive microglia were first to be identified before further infiltrating iNOS-positive monocytes were found (Figure 1A,B). The increase in iNOS+ microglia and inflammatory monocytes was significant in brains and TGs at 7 d p.i. (*p* ≤ 0.01) (Figure 1C).

Comparison of iNOS expression with the expression of pro-inflammatory cytokines (TNF-α and IL-1β) and interferons—IFN-α and IFN-γ (Figure 1D) in different parts of HSV-1-infected brains showed that iNOS correlated with high expression of TNF-α and IL-1β in the cortex and cerebellum (*p* ≤ 0.05) (Figure 1D). The proportions of pro-inflammatory cytokines, iNOS and viral titers were disadvantageous for the cerebellum, where we detected very high HSV-1 titers, followed by a high expression of pro-inflammatory TNF-α and IL-1β (*p* ≤ 0.01) (Figure 1E). IFN-α and IFN-γ showed very high expression in the cortex (*p* ≤ 0.01) (Figure 1D). The correlation between high expression of pro-inflammatory cytokines, iNOS and high HSV-1 titers resulted in the observed problems with movements and hunched backs, defined as clinical signs of encephalitis.

### 3.2. Inhibition of NO Production Induces Different Effects upon Response to HSV-1 Infection Depending on the Time of Treatment

**Figure 2:**
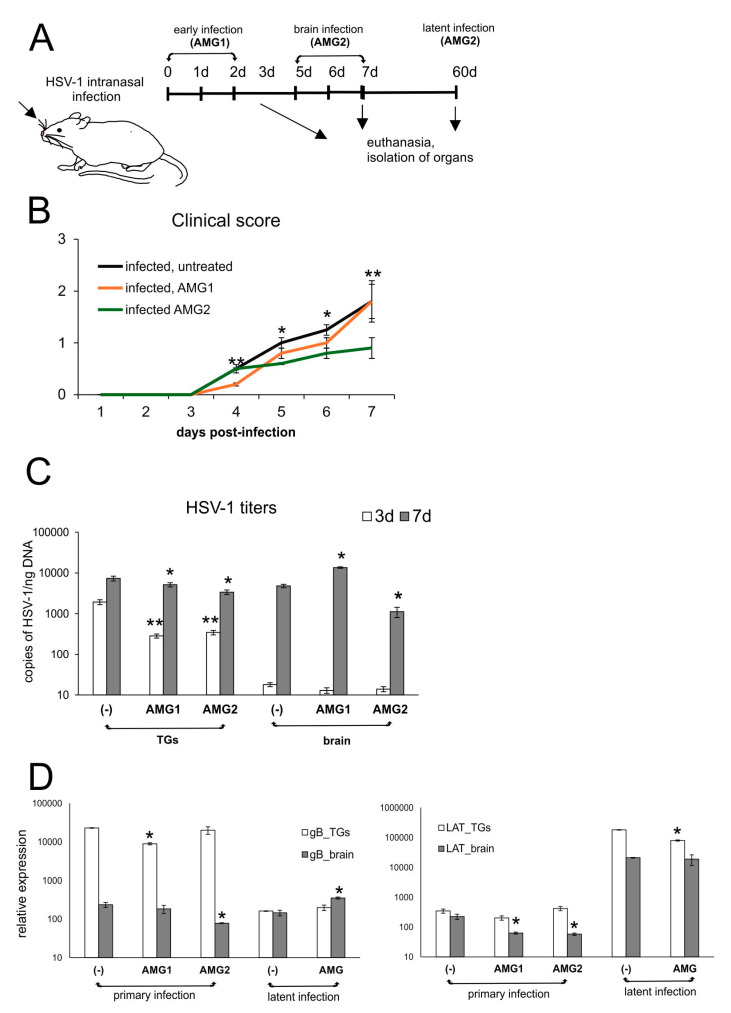
Inhibition of iNOS diminishes HSV-1 infection in vivo depending on the time of treatment. (**A**) C57BL/6 mice infected intranasally with HSV-1 were treated with AMG at 400 mg/kg daily either early during infection (AMG1) or late during infection (AMG2). (**B**) Clinical score of mice treated with AMG at different time points (neurological symptoms; see Materials and Methods). (**C**) HSV-1 viral titers and (**D**) LAT and gB mRNA viral transcripts in trigeminal ganglia (TGs), and brains collected at 3 and 7 days p.i., measured by qPCR (N = 7). The bars represent means ± SEMs. * represents significant differences with *p* ≤ 0.05, and ** represents *p* ≤ 0.01, in comparison to untreated infected tissues.

To assess how the inhibition of NO will influence HSV-1 infection in vivo, the mice were given aminoguanidine (AMG), an iNOS inhibitor, either early during infection (AMG1, days 1 and 2 p.i.) or during brain infection (AMG2, days 5 and 6 p.i.) (Figure 2A). We observed that the administration of the iNOS inhibitor improved overall clinical outcomes, although the administration of AMG during the peak of brain infection (days 5–7 p.i.) resulted in significantly better clinical outcomes (*p* ≤ 0.01) (Figure 2B), compared to early administration of AMG (days 1–2 p.i.). To understand how the inhibition of iNOS influences HSV-1 replication, we collected samples of brains and trigeminal ganglia on day 3 (peak infection of trigeminal ganglia, before infection of the brain), day 7 (peak of brain infection with neurological symptoms) and day 60 (latent infection). We found that AMG-treated mice showed significantly lower HSV-1 titers in TGs during primary infection at 3 and 7 d p.i. (*p* ≤ 0.05) (Figure 2C), irrespective of the treatment time, indicating the protective AMG effect upon early peripheral HSV-1 infection. In contrast, brains of mice treated early (AMG1) demonstrated significantly higher doses of HSV-1 (*p* = 0.03) (Figure 2C), while late treatment (AMG2) resulted in significantly lower HSV-1 titers at 7 d p.i. (*p* = 0.02). No significant differences were observed for latent infection at 60 d p.i. both for TGs and brains.

Measurement of viral gene expression (gB and LAT) showed that mRNA for structural gB protein followed HSV-1 DNA titers only during primary TGs upon AMG2 treatment and generally during latent infection (*p* ≤ 0.05) (Figure 2D). Concerning expression of the latency transcript (LAT) in AMG-treated mice, we observed that both times of AMG treatment resulted a significant decrease in LAT expression in brains but not in TGs (*p* ≤ 0.05) (Figure 2D), indicating better control of the virus by the immune system. Similar results were observed for TGs: treatment with AMG (1, 2) resulted in lower LAT mRNA levels in latently infected TGs (*p* = 0.05) (Figure 2D).

**Figure 3:**
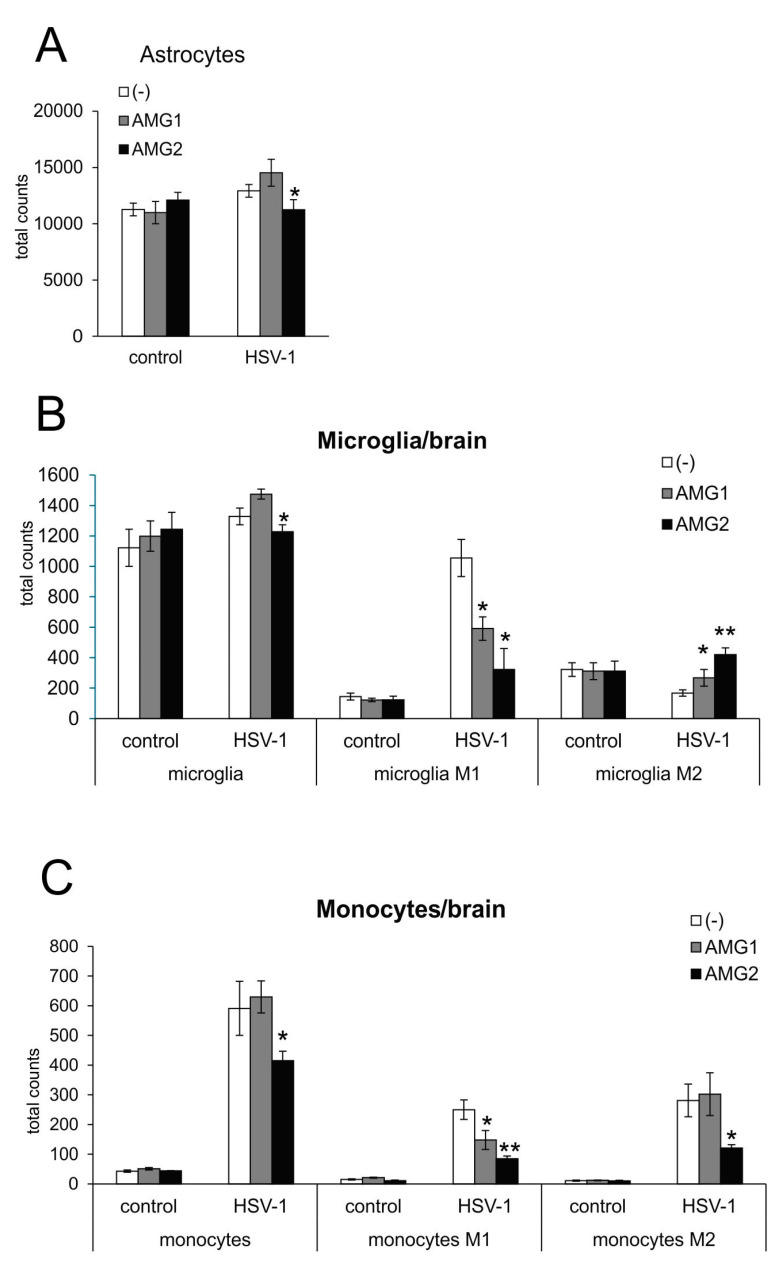
Inhibition of iNOS affects innate antiviral response during HSV-1 infection, depending on the treatment time. HSV-1-infected C57BL/6 mice were treated with AMG at 400 mg/kg daily either early during infection (AMG1) or late during infection (AMG2). Brains were collected at 7 days p.i. and subjected to the preparation of single-cell suspensions, further stained with appropriate antibodies and analyzed by flow cytometry. Total counts of (**A**) astrocytes, (**B**) microglia and microglia with an M1 or M2 phenotype and (**C**) monocytes and monocytes with an M1 or M2 phenotype in brains at 7 days post-infection by flow cytometry. Results are expressed as the mean ± SEM for N = 7. * represents significant differences with *p* ≤ 0.05, while ** signifies *p* ≤ 0.01, in comparison to HSV-1-infected, untreated tissues.

Astrogliosis and microgliosis are typical signs of ongoing neuroinflammation in HSV-1 infection. While early treatment with AMG increased the number of astrocytes, albeit insignificantly (*p* = 0.051) (Figure 3A), later treatment with AMG led to a significant decrease in astrocyte numbers (*p* = 0.02) (Figure 3A). The numbers of both microglia (Figure 3B) and infiltrating monocytes (Figure 3C) in the HSV-1-infected brains were significantly increased (*p* ≤ 0.05), and only later treatment with AMG led to a significant decrease in the numbers of both microglia and inflammatory monocytes compared to untreated infected brains (*p* ≤ 0.05) (Figure 3B,C).

Monocytes as well as microglia exhibit various types of activated phenotypes, referred to as classical activation phenotypes (M1), typically releasing pro-inflammatory mediators, and alternative activation phenotypes (M2), which possess anti-inflammatory properties [20,28]. The expression of iNOS is a marker characteristic for the M1 active phenotype. Evaluation of microglia phenotypes by flow cytometry showed that treatment with AMG significantly decreased the numbers of microglia and monocytes with the M1 phenotype (*p* ≤ 0.05) (Figure 3B,C), irrespectively of the treatment time. AMG treatment increased the numbers of anti-inflammatory M2 microglia in the brains, both in early and late treatments (*p* ≤ 0.05) (Figure 3B,C). Late treatment with AMG decreased M2 monocytes (*p* = 0.03) (Figure 3C).

**Figure 4:**
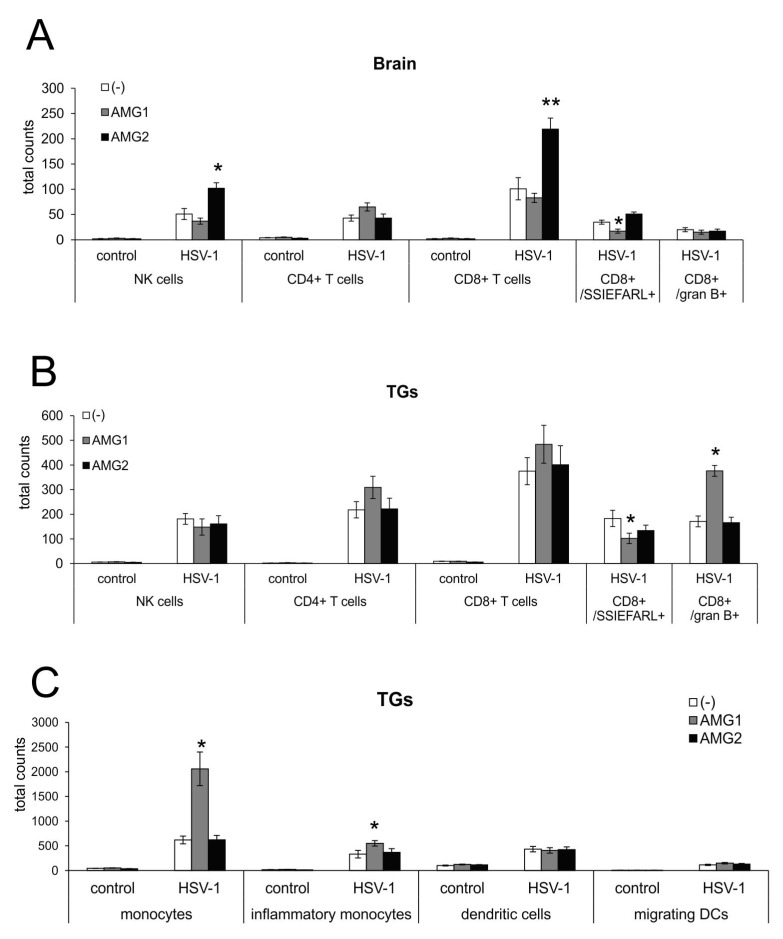
Time of NOS2 inhibition has opposite effects on antiviral response during HSV-1 infection. Total counts of NK cells, CD4+ T cells, CD8+ T cells, CD8+/SSIEFARL+, CD8+/granzyme B+ and T cells in brains (**A**) and TGs (**B**). (**C**) Total counts of monocytes, inflammatory monocytes, tissue-specific dendritic cells and migrating dendritic cells in TGs. HSV-1-infected C57BL/6 mice were treated with an inhibitor of iNOS, AMG, at 400 mg/kg daily either early during infection (AMG1) or late during infection (AMG2). Trigeminal ganglia (TGs) and brains were collected at 7 days p.i. and subjected to analysis by flow cytometry. Results are expressed as the mean ± SEM for N = 7. * represents significant differences with *p* ≤ 0.05, while ** signifies *p* ≤ 0.01 in comparison to HSV-1-infected, untreated tissues.

To determine how the inhibition of iNOS may further influence the development of an antiviral response in HSV-1 infection, we performed flow cytometry analysis of NK cells, CD4+ T cells, CD8+ T cells in the brain and TGs at 7 days of infection (Figure 4). We observed that early AMG treatment decreased CD8+ T cells/SSIEFARL+ numbers in the brains and TGs of mice treated early with AMG (*p* ≤ 0.05) (Figure 4A,B), compared to untreated infected mice. This was reflected by higher viral titers in the brain, as shown in Figure 2B. For the AMG1 treatment regime, only CD8+/granzyme B+ T cells were significantly increased (*p* = 0.034) (Figure 4B). In contrast, late treatment with AMG significantly increased the numbers of NK-1 cells and T cells in the brain (*p* ≤ 0.05), while other cells showed values similar to untreated infected organs (Figure 4A,B).

Interestingly, early treatment with AMG significantly increased the numbers of monocytes and inflammatory monocytes in trigeminal ganglia isolated at day 7 from AMG-treated, HSV-1-infected mice (*p* ≤ 0.01) (Figure 4C). Therefore, early inactivation of monocytes results in a further increase in inflammation within the peripheral nervous system. The inhibition of iNOS had no significant influence on the numbers of migrating and tissue-specific dendritic cells (DCs) in trigeminal ganglia at day 7 (Figure 4C).

**Figure 5:**
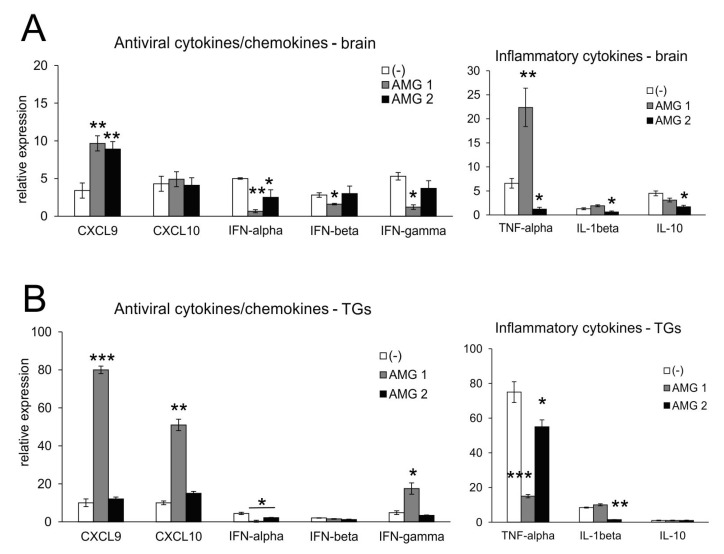
Inhibition of iNOS leads to changes in the production of cytokines and chemokines in the HSV-1-infected brains and TGs. Antiviral interferons (IFN-α,β,γ), chemokines (CXCL9 and CXCL10) and inflammatory cytokines (TNF-α, IL-1β and IL-10)) in the brains (**A**) and TGs (**B**) at 7 days post-infection were analyzed by qPCR. HSV-1-infected C57BL/6 mice were treated with an inhibitor of iNOS, AMG, at 400 mg/kg daily either early during infection (AMG1) or late during infection (AMG2). Trigeminal ganglia (TGs) and brains were collected at 7 days p.i. and subjected to analysis. Data are presented as the mean ± SEM, N = 7. *** represents significant differences with *p* ≤ 0.001, ** represents *p* ≤ 0.01, and * represents *p* ≤ 0.05 in comparison to HSV-1-infected, untreated tissues.

Microglia, astrocytes and other infiltrating immune cells contribute to viral immunity in the CNS via the production of IFNs and other antiviral and/or inflammatory cytokines/chemokines [23,24]. The effect of AMG upon cytokine/chemokine production was different depending on the timing. Aminoguanidine treatment in general increased the levels of mRNAs for CXCL9 in the brain (*p* ≤ 0.05) (Figure 5A) and mRNAs for CXCL9 and CXCL10 in TGs after early treatment (AMG1) (*p* ≤ 0.01) (Figure 5). High levels of CXCL9 and CXCL10 reflected significantly increased IFN-γ mRNAs in TGs (*p* = 0.01) (Figure 5B). For the brain, early treatment led to a significant decrease in IFNs (*p* ≤ 0.01) (Figure 5A), while late treatment with AMG resulted in the preservation of IFNs levels. Additionally, late AMG treatment reduced mRNA levels for inflammatory cytokines: TNF-α and IL-1β (*p* ≤ 0.01), especially in the brain (Figure 5A), and TNF-α in TGs (*p* = 0.001) (Figure 5B). For TGs, AMG2 treatment significantly reduced the mRNA levels for IL-1β and TNF-α (*p* ≤ 0.05) (Figure 5B).

### 3.3. NO Exerts Cell Type-Dependent Effects for Glial Cells upon HSV-1 Infection

**Figure 6:**
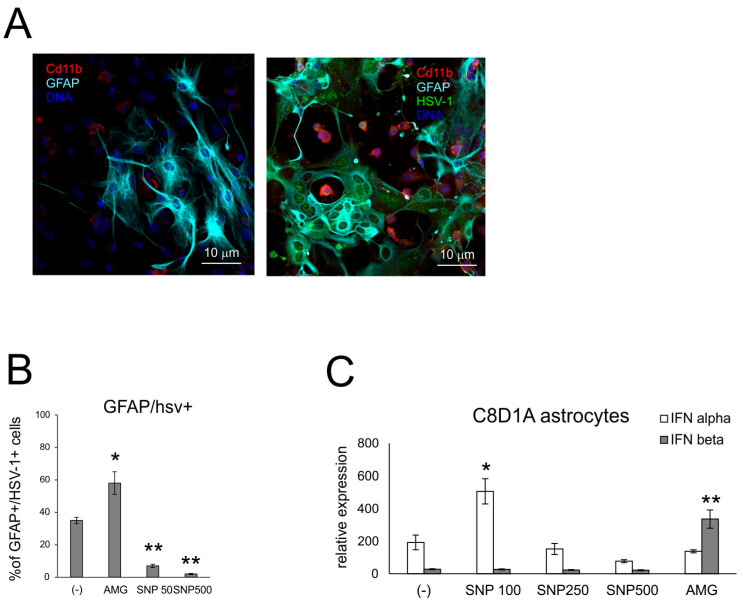
NO influences HSV-1 replication in glial cells. (**A**) Confocal microphotographs of uninfected (**left**) and HSV-1-infected (**right**) mixed glial cultures at 24 h p.i.; co-immunofluorescent staining for HSV-1 antigens (green), CD11b+ microglia (red) and GFAP+ astrocytes (turquoise). Nuclei were counterstained with DAPI (blue). Magnification × 200. (**B**) Percentage of HSV-1+ astrocytes (HSV-1+/GFAP+) in mixed glial cultures measured by flow cytometry. (**C**) C8D1A astrocytes were treated with AMG or SNP as given above and then infected in the same manner with HSV-1. After 24 h, cells were subjected to measurement of mRNA for IFN α and β with qPCR. Each bar represents the mean from 3 experiments (N = 3) ± S.E.M.; * represents significant differences with *p* ≤ 0.05, while ** signifies *p* ≤ 0.01 in comparison to infected cells.

**Figure 7:**
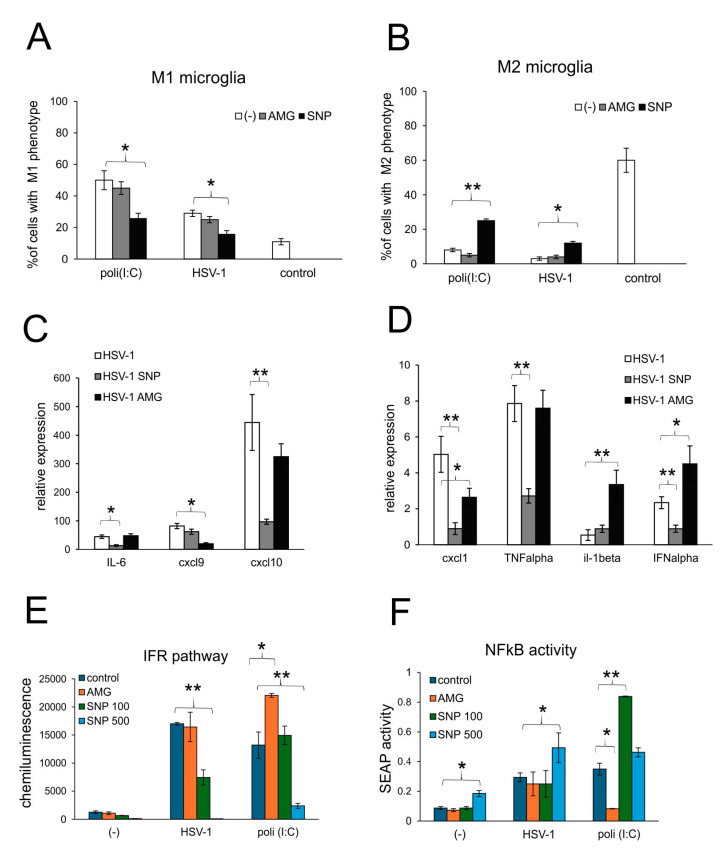
Nitric oxide regulates production of antiviral and inflammatory chemokine/cytokine during HSV-1 infection of glial cells in vitro. (**A**) M1 and (**B**) M2 phenotypes of microglia and (**C**,**D**) expression of IL-1α, TNF-α, IL-6, CXCL1, CXCL9, CXCL10 and IFN-α mRNA at 24 h p.i. in mixed glial cultures prepared from wild-type (C57BL/6) mice, untreated or treated with 50 μM AMG or 100 μM sodium nitroprusside (SNP) and measured with qPCR. (**E**,**F**) The RAW cells were treated with 50 μM AMG or 100, 250 and 500 μM sodium nitroprusside (SNP100, 250 and 500, respectively) and then left uninfected, infected with HSV-1 (1 PFU/cell) or treated with poly (I:C) at 1 μg/mL. After 18 h, the interferon regulatory factor (IFR) pathway (**A**) was measured as Lucia chemiluminescence, and the activation of the NF-κB pathway was measured as SEAP activity (**B**). Data from three independent experiments are presented as the mean ± SEM. Two-way ANOVA test was used. * represents *p* ≤ 0.05, while ** represents *p* ≤ 0.01 in comparison to untreated control, HSV-1 infection or poli (I:C).

We employed the mixed glial cultures to understand how NO influences the activity of microglia and astrocytes within the HSV-1-infected site (Figure 6 and Figure 7). Sodium nitroprusside (SNP) was used as an exogenous NO donor in the concentration range of 100–500 μM, and AMG at a concentration of 50 μM was used as an inhibitor of iNOS. Exogenous NO caused a significant decrease in the HSV-1 titers in mixed glial cultures (Supplementary Figure S1A), while AMG had no influence on HSV-1 replication (*p* ≤ 0.05) (Supplementary Figure S1). A significant decrease of HSV-1+ cells was observed for astrocytes in SNP-treated cultures (Figure 6B), from 38.2 ± 2.1 to 7.01 ± 0.89% of GFAP+/HSV-1+ cells, respectively (*p* ≤ 0.05) (Figure 6B), while no effect was observed for microglia. Treatment with AMG led to a significant increase in HSV-1+ astrocytes (*p* = 0.05) (Figure 6B). We also measured IFNα/β mRNA expressions in the HSV-1-infected C8D1A astrocyte cell line subjected to SNP and AMG during HSV-1 infection (Figure 6C). For astrocytes, IFNα mRNA expression was dose dependent, with low NO concentration (100 uM) inducing IFNα expression. IFNβ expression was only induced upon AMG treatment (*p* ≤ 0.05) (Figure 6C).

The production of NO is attributed to an activated M1 phenotype of microglia and monocytes [26,27], acting through a paracrine manner. To further determine how nitric oxide may regulate the active phenotype of microglia during HSV-1 infection, we determined the percentage of M1 and M2 phenotypes in HSV-1-infected mixed glial cultures (Figure 7A,B). Microglia from uninfected cultures showed significantly more M2 phenotype cells (*p* ≤ 0.05) (Figure 7A,B). HSV-1 infection induced a significant increase of M1 to M2 proportion (*p* ≤ 0.05) (Figure 7A,B), although two times smaller than poli (I:C) (*p* ≤ 0.05) (Figure 7A,B). The addition of NO from an external source led to the significant decrease in the percentage of M1 microglia in poli (I:C)-treated cultures and a significant increase in M2 microglia (*p* ≤ 0.05) (Figure 7A,B). This effect of NO was also observed in HSV-1-infected cultures, indicating that NO can regulate the activation of TLR3-stimulated microglia via a back-loop mechanism (Figure 7A,B).

Activation patterns of microglia were reflected by the profile of cytokine and chemokine production (Figure 7C,D). Mixed glial cultures treated with exogenous NO significantly down-regulated the expression of mRNA for IFN-α, TNF-α, IL-6, CXCL1 and CXCL10 (*p* ≤ 0.05) (Figure 7C,D). The expression of CXCL9 remained unchanged upon infection, and only the inhibition of INOS with AMG resulted in a significant down-regulation of mRNA (*p* = 0.022) (Figure 7C,D). Treatment with AMG led to the up-regulation of IFN-α and IL-1β (*p* ≤ 0.05) (Figure 7C,D).

Next, we assessed how NO influences NF-κB signaling, the critical inflammation transducer in HSV-1 infection of monocytes. NF-κB signaling was tested using RAW-Dual™ cells, which allow us to simultaneously study the NF-κB pathway and the interferon regulatory factors (IFR) pathway. RAW-Dual™ cells were subjected to an NO donor, SNP, or iNOS inhibitor, AMG, and infected with HSV-1. At 18 h p.i., cells were monitored for NF-κB and IFR pathways. The results showed that NO decreased IFR pathways in a dose-dependent manner for both HSV-1 and poli (I:C), a TLR3-dependent inducer of the IFR pathway (*p* ≤ 0.05) (Figure 7E). An opposite effect was observed for the inhibition of iNOS activity, resulting in the activation of a TLR3-dependent IFR pathway (Figure 7E). NO induced NF-κB signaling in all experimental settings, although in a dose-dependent manner, with high doses effective for control and HSV-1-infected cells (*p* ≤ 0.05) (Figure 7F). Inhibition with AMG decreased TLR3-dependent NF-κB signaling (Figure 7F).

### 3.4. Inhibition of NO Production Early During Infection Influences HSV-1-Related Neurodegenerations Markers

**Figure 8:**
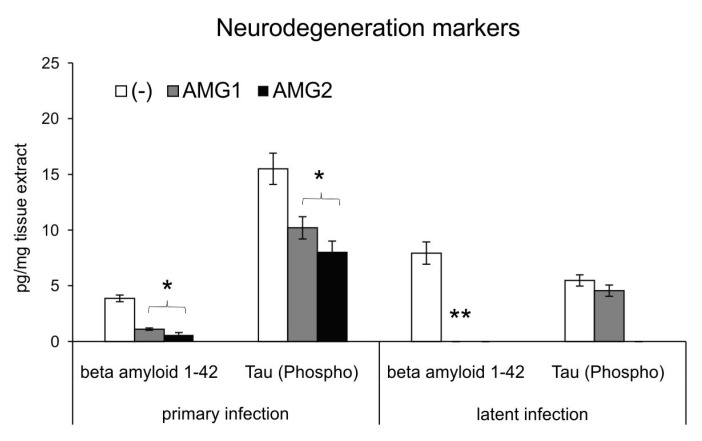
Inhibition of NO production early during infection influences HSV-1-related neurodegenerations markers. HSV-1-infected C57BL/6 mice were treated with an inhibitor of iNOS, AMG, at 400 mg/kg daily late during infection (AMG2). Brains were collected at 7 (primary infection) and 60 days (latent infection) p.i. and used to prepare brain extracts used to quantify beta amyloid 1–42 and Tau (Phospho) [pS199] by ELISA. Results are expressed as the mean ± SEM for N = 7. * represents significant differences with *p* ≤ 0.05, and ** means *p* ≤ 0.01, in comparison to HSV-1-infected, untreated tissues.

Neuroinflammation is now well recognized as an important pathological process leading to neurodegenerative diseases of CNS [17] and a potential target for therapy and prevention. Microglia can play a dual role in the pathogenesis of neurodegeneration. Activated M1 microglia mediate an inflammatory response, leading to the accumulation of damaged or misfolded proteins, such as phosphorylated Tau and beta-amyloid [17,18,19,20,21,22], responsible for the destruction of the brain tissue and further cognitive and functional sequelae. Here, we measured the concentrations of Tau (Phospho) [pS199] and 1–42 beta amyloid in extracts obtained from the brains of HSV-1-infected mice treated and untreated with the iNOS inhibitor AMG both early and late during infection. We found that mice treated during infection with AMG show significantly lower levels of phosphorylated Tau and beta 1–42 amyloid peptides at day 7 p.i. (*p* ≤ 0.05) (Figure 8). During the latent phase of infection, AMG-treated mice showed significantly lower levels of beta 1–42 amyloid peptides at day 60 post-infection (*p* ≤ 0.05) (Figure 8).

### 3.5. Inhibition of NO Production During HSV-1 Decreases Fas/FasL-Dependent Inflammation but May Increase Viral Titers

We previously discussed the role of Fas/FasL during HSV-1 infection and showed that excessive Fas/FasL-dependent inflammation is responsible for the observed neuroinflammation during the peak of infection [31] Here, we decided to check first if we can observe any correlation between NO donor/inhibitor and the presence of a functional Fas/FasL pathway. When using mixed glial cells prepared from mice lacking either Fas-B6. MRL-Fas lpr/J or FasL -B6Smn.C3-Fasl gld/J (FasL), we found no differences between wild-type and Fas- or FasL-deficient strains (*p* > 0.05) (Supplementary Figure S1A). Therefore, we tested if the AMG treatment can influence the numbers of FasL+ positive cells during in vivo infection (Supplementary Figure S1B). Our tests demonstrated that late treatment with AMG significantly decreased the numbers of FasL+ CD8+ T cells, inflammatory monocytes and microglia, which has been demonstrated to participate in Fas/FasL-dependent neuroinflammation (*p* ≤ 0.05) (Supplementary Figure S1B). No effects were observed upon early AMG treatment (Supplementary Figure S1B). In the next step, we applied HSV-1 infection and AMG treatment to mice lacking either Fas or FasL (Supplementary Figure S1C). We found that while Fas (−) and FasL (−) mice showed significantly lower titers of HSV-1 both in the brains and TGs at 7 d p.i., AMG treatment early during infection resulted in increased titers in the brains of Fas- and FasL-deficient mice compared both to untreated controls but also to AMG-treated wild-type mice (*p* ≤ 0.05) (Supplementary Figure S1C). For TGs, early AMG treatment led to a significant decrease in HSV-1 titers for all tested strains (*p* ≤ 0.05) (Supplementary Figure S1C). No differences for viral titers were found when AMG treatment was applied late in infection.

## 4. Discussion

Our present study discusses the role of NO in response to HSV-1-infection as a modulator of early antiviral innate immunity and as a causative agent of prolonged neuroinflammation and neurodegeneration. The role of NO in viral infections is complex: while NO has a positive direct antiviral effect on some viruses, it may still have negative effect upon development of the further antiviral response. Direct inactivation of virions was demonstrated for influenza virus and SARS-CoV-1 [10,11,35,36], while for rhinovirus and parainfluenza virus, NO inhibited viral replication and the production of pro-inflammatory cytokines [9,37,38]. NO can also inhibit the membrane fusion of the virus by interfering with the interaction between the SARS-CoV-1 viral proteins and the membrane receptor [35,36]. Similar results were observed in this study for a decrease in replication of HSV-1-infected astrocytes, accompanied by an increase in the production of antiviral IFN-α, although this effect was clearly dose dependent.

In contrast to respiratory viruses, NO production during non-respiratory infections is mostly associated with pathogenic effects due to inflammation. In chronically infected patients with hepatitis C virus (HCV), there is a strong positive correlation between iNOS expression and the level of viral RNA and tissue damage, suggesting a role for NO in the pathogenesis of chronic viral hepatitis [13]. During dengue virus infection, NO, as an inflammatory mediator with antiplatelet and anticoagulant actions, is involved in vascular and platelet dysfunction in dengue, culminating in plasma leakage and hemorrhage [39,40]. For neuronal infections like rabies, NO creates peroxynitrate, which further damages the blood–brain barrier through lipid peroxidation [41]. The use of AMG as iNOS inhibitor in rabies virus infection of mice led to increased survival time, a delay in rabies clinical signs, reduced viral load and histopathological lesions in mice, suggesting the role of NO in rabies virus replication [41].

Here, treatment of animals with an inhibitor of iNOS early during infection HSV-1 infection impaired the antiviral response, leading to higher virus titers in the brain. On the other hand, the inhibition of iNOS during the peak of brain infection led to better clinical outcomes, lower viral titers and lower neuroinflammation. This confirms previously published data showing higher mortality rates if iNOS was inhibited before HSV-1 infection [28,42]. Furthermore, the inhibition of iNOS at the peak of infection decreased neurological symptoms and increased survival of infected animals [43,44]. Hasson et al. also demonstrated that HSV-1 inhibits the activation of brain NOS, which leads to neuronal viral invasion [45]. This suggests that NOS activity is involved in the early stages of the viral infection, acting as antiviral agent, while later it may participate in pathogenesis of neuroinflammation.

The main sources of NO during viral infection are macrophages, neutrophils, monocytes and endothelial cells stimulated by IFN-γ [8]. Zolini et al. (2014) demonstrated that macrophage response (NO production) is necessary for controlling the immune response in the TGs of HSV-1-infected mice [27]. Lucinda et al. (2017) confirmed that monocytes and macrophages were the primary iNOS-producing cells in the TGs, and iNOS is essential in mediating the local (TG), but not systemic, immune response against HSV-1 [46].

In this study, early treatment with AMG ended at day 3, when the first HSV-1 antigens are normally detected in the brain [32], meaning that our AMG1 treatment affected mostly infection of TGs. Even though early inhibition of iNOS leads to a decrease in IFNα/β expression, which is further reflected by the decreased numbers of HSV-1-specific CD8+ T cells in TGs, it was also accompanied by the infiltration of inflammatory monocytes, leading to virus elimination by monocyte-dependent mechanisms. Nevertheless, protection of the brain from HSV-1 infection clearly requires the production of NO in TGs, as shown previously [27,46].

Furthermore, the local dose of NO seems to be crucial for the observed effects of NO in monocytes. Exogenous NO decreased the interferon response pathway (IFR) in a dose-dependent manner for both HSV-1 and poli (I:C), a TLR3-dependent inducer of IFR pathway, which may indicate the existence of a back-loop mechanism, serving as stop signal upon viral infection. This may be also a sign of a virus-induced evasion mechanism. At the same time, high doses of NO induced NF-κB signaling related to toxicity and inflammation.

The dose-dependent effects of NO upon cellular response have been demonstrated before. NO can affect T-cell response, with elevated concentrations inhibiting the immune system and disrupting IL-2R signaling by inhibiting the activation of Janus kinases and STAT5 [47,48]. On the other hand, low concentrations of NO stimulate the generation of the secondary messenger cyclic guanosine monophosphate (cGMP), thereby promoting Th1 (CD4+ and CD8+ T cell) differentiation but not Th2 [49].

Under physiological conditions, microglia exist in a resting state, and activated microglia have two states, the pro-inflammatory M1 type and the anti-inflammatory M2 type [23,50]. The M1 state is characterized by the production and secretion of pro-inflammatory cytokines (IL-1β, IL-6 and TNF-α), ROS and NO, whereas M2 microglia facilitate resolution of inflammation through anti-inflammatory factors (IL-10 and TGF-β) [23,50]. It is known that M1 macrophages are refractory to HSV-1 replication, while M2 macrophages promote viral replication [50]. Here, we found that high exogenous NO in HSV-1-infected microglia in vitro decreases M1 activation and the production of antiviral chemokines CXCL9, CXCL10 and IFN-α in co-cultures with astrocytes. Activated microglia can control HSV-1 replication in the brain by producing inflammatory cytokines and chemokines such as IL-6, IL-1b, type I interferons (IFN-I), CXCL-10, CCL2 and CCL5, which activate other glia cells within the CNS as well as attract immune cells, such as DCs, NK cells and T cells, to the brain [23,51]. In other words, microglia play an important role in mounting of the adaptive immune response in the CNS. The pharmacological depletion of microglia was shown to increase brain viral loads and mortality in mouse models of West Nile virus encephalitis and mouse hepatitis virus encephalitis, suggesting that the initiation of the innate and adaptive immune responses requires functional microglia [52,53].

Since HSV-1 infection of the brain suppresses the activity of constitutive cNOS [44], the infiltrating monocytes remain the main source of NO, together with activated microglia. Furthermore, at the peak of HSV-1 infection in the brain, iNOS expression correlates with the production of inflammatory cytokines (TNFα and IL-1β), confirming the involvement of NO in HSV-1-induced neuroinflammation and deregulation of the local immune response. The inhibition of iNOS late in the infection returned the inflammatory reaction to normal, followed by the production of antiviral cytokines, infiltration of cytotoxic CD8+ T cells, lower viral titers and, eventually, better clinical outcomes. Similar results were obtained by Madhu et al. for rabies in mice: AMG treatment resulted in increased numbers of CD4+, CD8+ and NK cells in the blood and spleen, accompanied by increased survival time, delayed development of clinical signs, reduced viral load and less apoptotic cells [41].

The deleterious role of NO in neuronal infections involving neuroinflammation was demonstrated for rabies [41], HIV [54] and dengue with CNS involvement [55].

Despite “the immune privilege of the brain,” neuroinflammation is now well recognized as a prominent feature in pathology of neurodegenerative diseases and a potential target for therapy and prevention of this disease [17,18,19,20,21,22,56].

Microglia and astroglia are consistently found surrounding amyloid plaques and neurofibrillary tangles in the brains of patients with Alzheimer’s disease (AD) [57,58,59,60,61,62], but microglia can play dual roles in AD pathogenesis. Microglia help eliminate β-amyloid aggregation via phagocytosis; on the other hand, amyloid deposition causes a microglial-mediated inflammatory response [57,58,59]. Many studies have strongly supported the concept that repeated reactivations of HSV-1 may lead to persistent neuroinflammation, the formation of amyloid plaques and neurofibrillary tangles and, eventually, to AD. Mouse brains infected with HSV-1 show increased β-amyloid deposition early after infection and cognitive impairment in a long-term, recurrent HSV-1 infection [60]. Neuroinflammation-induced cell death is often derived from the long-term impact caused by the increase in reactive oxygen and nitrogen species, which play a major role in eliciting apoptotic cell death through irreversible oxidative or nitrosative injury to neurons [57,58,59,60,61,62].

In this study, we found that treatment of mice with AMG led to significant decrease in the levels of 1–42 beta amyloid both during primary and latent infections, but it also reduced the levels of Tau (Phospho) [pS199], a component of neurofibrillary tangles characteristic for neuroinflammation [57,58,59,60,61,62]. This effect could contribute to an AMG-induced decrease in microglial activation (despite high virus titers) but also to the properties of AMG itself. AMG is a potent antioxidant compound and free radical scavenger [30,63]. It is a potent scavenger of peroxynitrite in addition to oxygen free radicals or metabolites [30,63]. Since AMG can cross the BBB, it is worth testing it in the treatment of long-term effects of neuroinflammation and accompanying damaging effects of oxidative stress. We propose to consider the possibility of using iNOS inhibitors during the peak of HSE to protect the patients from possible sequelae of HSE, such as mental retardation, neurological complications and autoimmune encephalitis.

Furthermore, since excessive production of NO has been demonstrated for many other viruses causing neuroinfectious and neuroinflammation, such as SARS-CoV-2, neurotropic flavivirus infections (tick-borne encephalitis virus, Zika virus, Japanese encephalitis virus and West Nile virus), but also by common measles virus and influenza virus [8,64], it is worth focusing on the possible uses of iNOS inhibitors, which can pass the BBB, to protect patients from the negative consequences of encephalitis. The role of NO in the encephalitis of each virus should be carefully investigated using both in vitro and in vivo models; however, our study confirms the link between iNOS inhibition and protection from neurodegeneration possibly observed later in life.

We have demonstrated previously that HSV-1 can interfere with Fas-mediated pro-inflammatory pathways within the CNS, leading to disturbances in Fas-mediated apoptosis and pro-inflammatory responses upon migration of FasL-bearing lymphocytes into the infected neuronal site [32]. The Fas (CD95 and APO-1) signaling pathway is a so-called receptor apoptotic pathway. Fas and FasL play critical roles in the immune system, being the direct mechanisms responsible for the killing of pathogen-infected target cells and the death of autoreactive lymphocytes [32]. Fas is not normally expressed on CNS cells; however, its expression can be induced within inflammatory sites, resulting in their susceptibility to FasL-induced death [32]. Within the CNS, FasL expression is detectable in neurons, microglia and perivascular astrocytes [65]. Previously, we found that NO decreases Fas expression on microglia while increasing FasL expression in HSV-1-infected glial cultures in vitro [31]. Here, we found that NO activity in HSV-1-infected glia is independent of Fas or FasL presence; however, the Fas/FasL pathway and NO may be indirectly linked with the antiviral response in vivo.

## 5. Conclusions

HSV-1 encephalitis (HSE) is a severe infection of the CNS with limited therapeutic options, which are not effective against the inflammatory components of HSE. Increasing evidence shows that uncontrolled neuroinflammation, even if the viral replication is low, is one of the primary sequelae of HSV-1 infection within the CNS, which could further lead to neurodegeneration process. Our study highlights the complex role of NO in HSV-1 infection, since its effects are dependent on the concentration, phase and site of infection (peripheral vs. central nervous system). At optimal doses and when acting on cells of TGs, NO has a protective and regulatory function; however, NO has toxic effects at higher concentrations and when acting within inflammatory sites of HSV-1 brain infection. HSV-1 may also interfere with the regulation of NO production, which further adds to development of excessive inflammation, related to neurodegenerative processes during latent infection. We found that treatment with the iNOS inhibitor AMG at the peak of brain infection can improve antiviral response and reduce negative effects of HSV-1-related neuroinflammation. Therefore, treatment with iNOS inhibitors creates a new therapeutical goal for virus-induced encephalitis.

## Data Availability

The original contributions presented in this study are included in the article/Supplementary Material. Further inquiries can be directed to the corresponding author.

## Supplementary Materials

The following supporting information can be downloaded at: https://www.mdpi.com/article/10.3390/microorganisms13102222/s1, Ref [66] is included in Supplementary Materials. Figure S1. (A) Viral loads were quantified using qPCR detecting gB gene in DNA extracted mixed glial cultures at 24 h post infection. (B) Total counts of FasL+ microglia, inflammatory monocytes , CD8+ T cells. (C) Viral copies of the gB gene in brains measured using qPCR, Figure S2. FACS gating strategy for brains and TGs. Table S1. The list of antibodies used in the study.

## Abbreviations

The following abbreviations are used in this manuscript:

AMG	aminoguanidine
SNP	sodium nitroprusside
TGs	trigeminal ganglia
NO	nitric oxide
