# Supplementary material for: The Role of Nitric Oxide in HSV-1 Infection: The Use of an Inducible Nitric Synthase Inhibitor Aminoguanidine to Treat Neuroinflammation
Source: Microorganisms. 2025 Sep 23;13(10):2222. doi: 10.3390/microorganisms13102222 (PMC12566453; doi:10.3390/microorganisms13102222)

Supplementary Figure S1

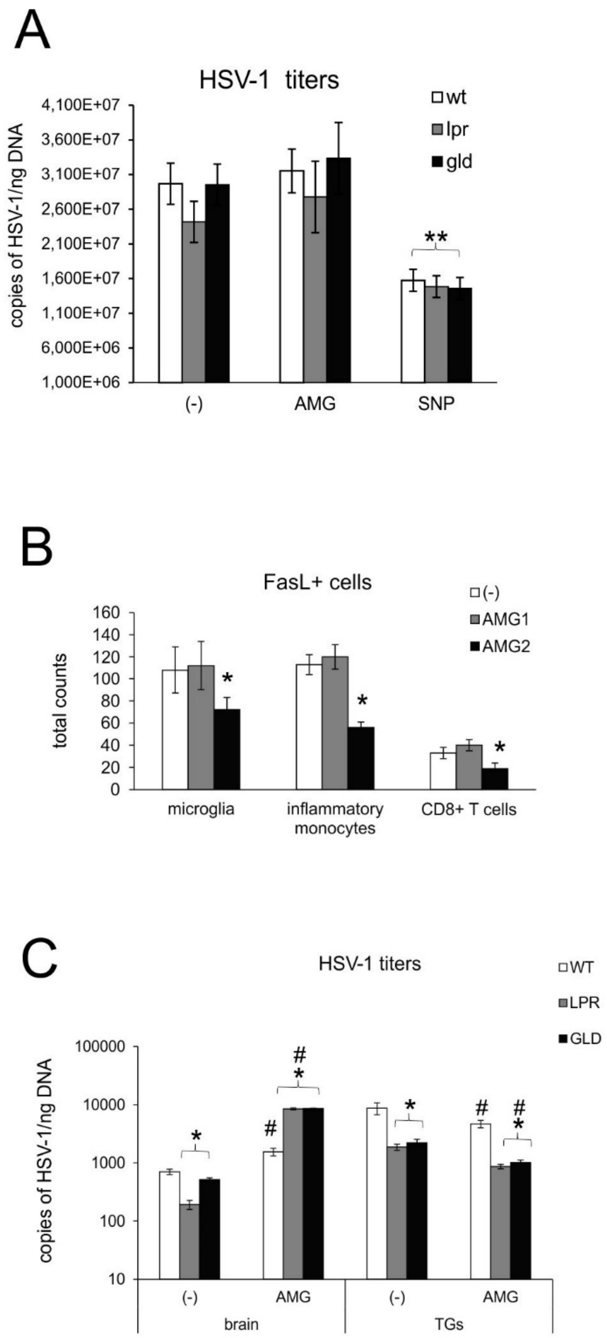

Supplementary Figure S1. (**A**) Viral loads were quantified using qPCR detecting gB gene in DNA extracted mixed glial cultures at 24h post infection, untreated or treated with 50 μM aminoguanidine sulphate (AMG) or 100 μM sodium nitroprusside (SNP). Mixed glial cultures were prepared from wild-type (C57BL/6), Fas-deficient (lpr) and FasL-deficient (gld) mice. n = 3 All data were presented as mean ± SEM. ** indicates P ≤ 0.001, compared to untreated control. (**B**)Total counts of FasL+ microglia, inflammatory monocytes , CD8+ T cells. HSV-1-infected C57BL/6 mice were treated with an inhibitor of iNOS, aminoguanidine sulphate at 400mg/kg daily either early during infection (AMG1) or late during infection (AMG2). Brains were collected at 7 days p.i., and subjected to analysis by flow cytometry. n =7 * indicates P ≤ 0.05, compared to untreated control. (**C**) Viral copies of the gB gene in brains measured using qPCR. C57BL/6 (WT), B6. MRL-Fas lpr/J (Fas−) and B6Smn.C3-Fasl gld/J (FasL−) mice were HSV-1 infected and

treated with AMG early during infection (AMG1). The virus titers were measured at day 7 p.i. n = 7. All data were presented as mean ± SEM. ** indicates P ≤ 0.001, while * P ≤ 0.05 compared to WT strain. ## indicates P ≤ 0.001, while # P ≤ 0.05 compared to untreated infected brains of each strain.

Supplementary Table S1

The list of antibodies used in the study

Antigen	Clone	Manufacturer
Anti-CD45-BV421 or APC-Cy7	A20	BD Biosciences
anti-CD3-FITC	145-2C11	ThermoFisher Scientific
anti-CD8-PE or BV421	53-6.7	BD Biosciences
anti- NK1.1-APC	PK136	BD Biosciences
anti-CD11b-PE	RB6-8C5	BD Biosciences
anti-CD192-BV421	475301	BD Biosciences
anti-Ly6C-APC-Cy7	AL-21	BD Biosciences
anti-IBA-1-FITC	EPR16588	Abcam
anti-CD86-PE	GL1	BD Biosciences
anti-CD206-APC	MMR	ThermoFisher Scientific
anti-FasL-PE	MFL3	BD Biosciences
anti-granzyme B-PE	NGZB	ThermoFisher Scientific

Materials and methods:

The followimg antibody combinations were used to detect immune cells: CD45/CD3/CD4/CD8 for helper (CD4+) T cells and cytotoxic (CD8+) T cells; CD45/CD3/NK1.1./CD4 for NK cells; CD45+/CD11b-/ or F4/80-/MHCII+CD11c+ for dendritic cells; CD103+/MHCII+CD11c+ for migrating dendritic cells; IBA1+/CD45 low/CD192- for microglia; CD45high/CD192+/CD11b+/Ly6C+ for infiltrating monocytes. Additionally, M1 microglia/monocyte were differentiated using additional CD86 antibody and M2 cells were identified as CD86-/CD206 high.

The compensation matrix was calculated using antibody capturing beads (VersaComp, Beckman Coulter, Poland) stained with single antibody conjugates. This approach was highly recommended by the manufactuter as it provided a higher level of standardization.

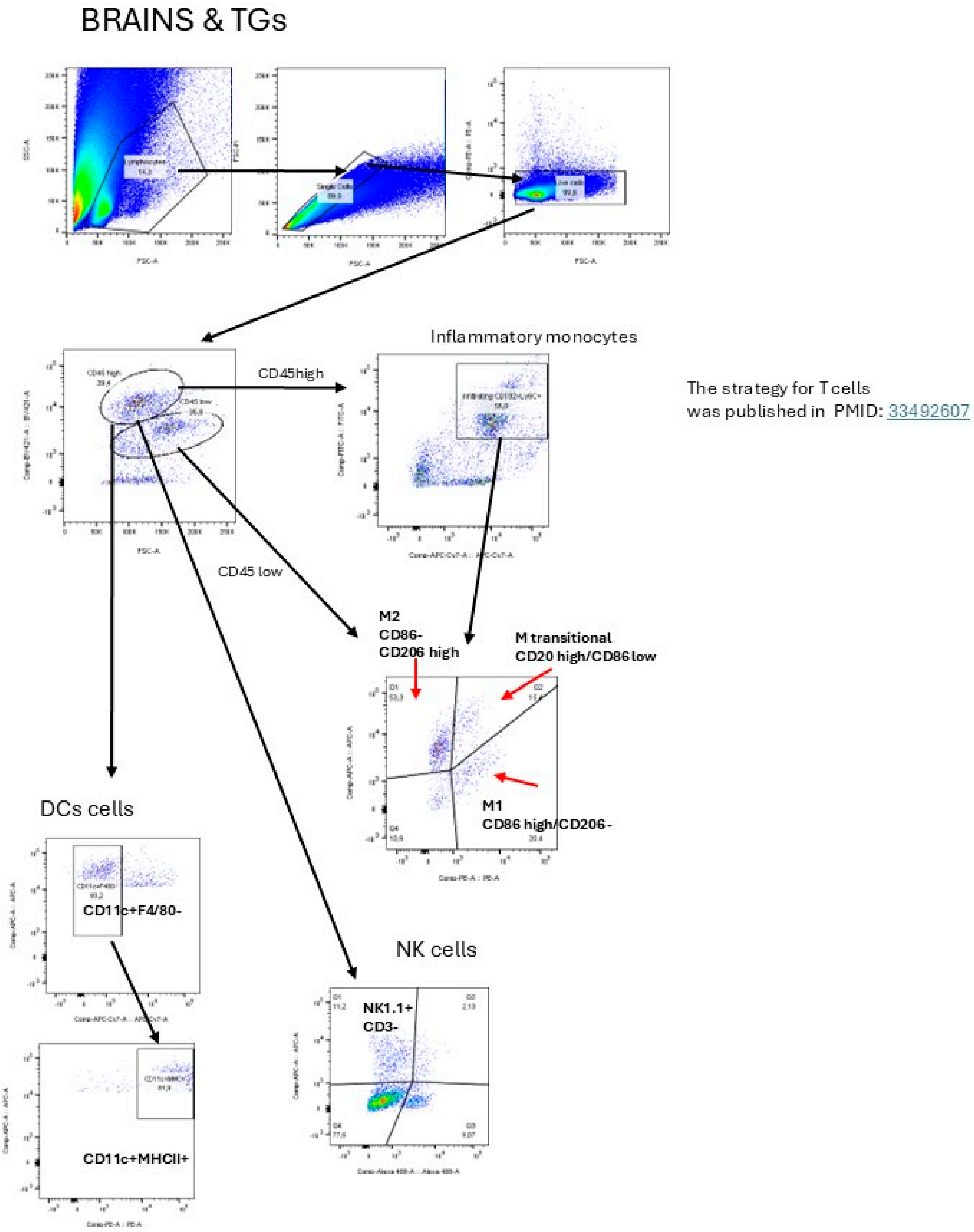

Supplementary Figure S2. FACS gating strategy for brains and TGs. The gating strategy for NK cells, DC cells, microglia and inflammatory monocytes is shown, while the gating strategy for T cells was published before: PMID: 33492607 [66].

Original microimages used in the paper:

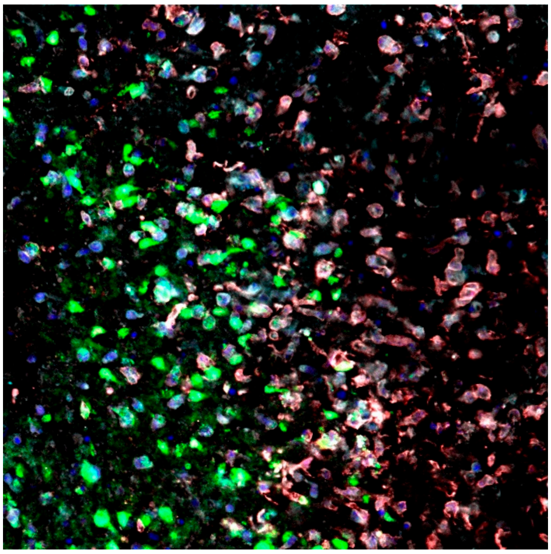

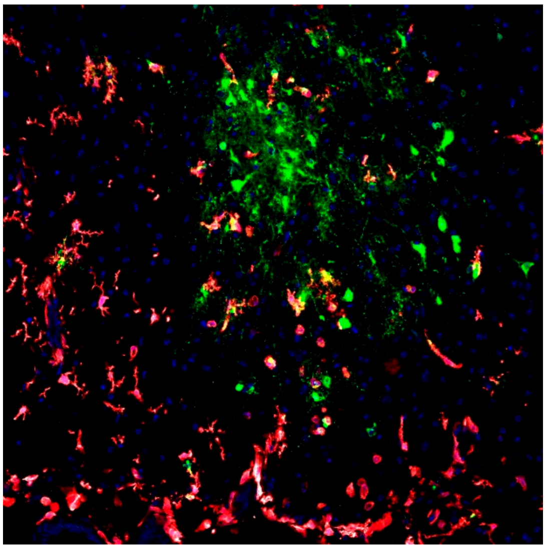

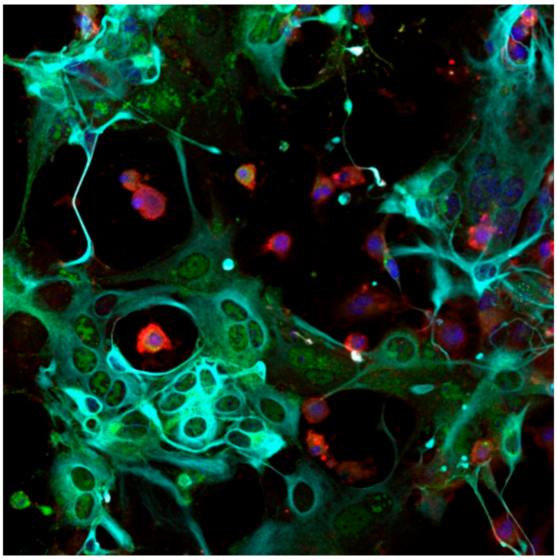

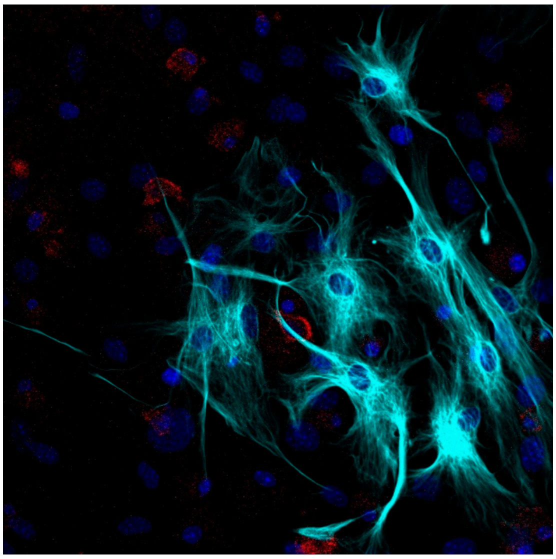

Supplement: Supplementary file 1 [file microorganisms-13-02222-s001.zip › microorganisms-3824682-supplementary.pdf]
